# Editorial: Deep learning techniques and their applications to the healthy and disordered brain - during development through adulthood and beyond

**DOI:** 10.3389/fnins.2022.1118233

**Published:** 2022-12-22

**Authors:** Amir Shmuel, Hyunjin Park, Yogesh Rathi, Albert Yang

**Affiliations:** ^1^Department of Neurology, McConnell Brain Imaging Centre, Montreal Neurological Institute, McGill University, Montreal, QC, Canada; ^2^Department of Neurosurgery, McConnell Brain Imaging Centre, Montreal Neurological Institute, McGill University, Montreal, QC, Canada; ^3^Department of Physiology and Biomedical Engineering, McConnell Brain Imaging Centre, Montreal Neurological Institute, McGill University, Montreal, QC, Canada; ^4^Department of Electrical and Computer Engineering, Sungkyunkwan University, Suwon, South Korea; ^5^Center for Neuroscience Imaging Research, Institute for Basic Science, Suwon, South Korea; ^6^Department of Psychiatry, Harvard Medical School, Boston, MA, United States; ^7^Department of Radiology, Harvard Medical School, Boston, MA, United States; ^8^Institute of Brain Science, National Yang-Ming Chiao Tung University, Taipei, Taiwan

**Keywords:** deep learning, medical image segmentation, neuroimaging-based diagnosis, Alzheimer's disease, Parkinson's disease, multiple sclerosis, schizophrenia, autism

Data acquisition methods used in medical imaging have been developing at an unprecedented pace; however, the interpretation of the data and their use for identifying and detecting biomarkers of disease can be difficult. The use of standardized computational aids has been proven very effective in overcoming this difficulty. Deep learning (DL) is a sub-group of machine learning algorithms capable of automatically extracting discriminatory features from raw input. This capacity makes DL a powerful technique that is already transforming neuroimaging data acquisition, modeling, and analysis. DL is a key player in MRI image reconstruction of sub-sampled *k*-space (Zeng et al., 2021), making it possible to reduce the duration of data acquisition. DL is capable of domain-specific image processing, with example applications in the retrospective correction of movement artifacts (Küstner et al., 2019) and monitoring and quality control of large MRI databases (Pizarro et al., 2019). Importantly, DL has been showing promise in the early diagnosis of several diseases and disorders, including autism spectrum disorder, Alzheimer's disease, and Parkinson's disease (Feng et al., 2022). The potential of DL to be useful also to basic research—not necessarily related to diagnosis—is high. The aim of this Research Topic is to highlight the potential of applying DL techniques to neuroimaging methods and applications.

The applicability of deep learning to a wide range of neuroimaging data acquisition methods is reflected in the type of data presented in the Research Topic, including structural MRI, diffusion MRI, EEG, and microscopy data. Similarly, deep learning is applicable to a wide range of neurological, and neurodevelopmental conditions studied by neuroimaging. Indeed, the Research Topic includes studies that apply deep learning to data on Alzheimer's disease, vascular cognitive impairment, Parkinson's disease, multiple sclerosis, autism, and neurodevelopment in pre-term infants possibly leading to cognitive deficits. The only psychiatric condition studied in this Research Topic is schizophrenia. However, thanks to its capacity to detect bio-markers that were not pre-defined, we expect that deep learning will be central to advancing biomarker identification of all psychiatric conditions.

Deep learning neural networks are especially capable of performing analysis of structured data, such as images and volumes. The Research Topic includes five studies that developed and/or evaluated deep learning methods for image segmentation. Brusini et al. **(ms. 469755) seek ways to improve the segmentation of the hippocampus** in structural MRI images. The structural integrity and volume of the hippocampus have been implicated as a biomarker in neurodegenerative conditions including Alzheimer's disease. They propose a DL-based hippocampus segmentation framework that embeds the statistical shape of the hippocampus as context information for learning. Their results suggest that adding shape information can improve the segmentation accuracy in cross-cohort validation, i.e., when deep neural networks are trained on one cohort and applied to another. **Another brain region implicated in neurological and psychiatric conditions is the amygdala**, whose segmentation is challenging due to its small dimensions. Large image patches are more likely to be dominated by background voxels, creating a class imbalance between the background class and the class of the small amygdala. Segmenting small structures such as the amygdala introduces a trade-off between capturing a sufficiently large context and retaining fine details while alleviating the imbalanced class issue. Alexander et al. **(ms. 497969)** addressed this challenging task by developing a dual-branch dilated residual 3D fully convolutional network (i.e., a network that performs only convolution, sub-sampling, and up-sampling) using receptive fields at the approximate size of the regions of interest with parallel convolutions to extract global context. **Segmenting the neonatal cerebrum according to tissue type is challenging** given its uniquely inverted tissue contrasts. Existing neuroimaging analysis packages are primarily designed to work on MRI with adult contrast but inversed water-to-cholesterol ratio in newborns leads to inverted MRI tissue contrast, hindering analyses. Ding et al. **(ms. 493147)** evaluated the performance of two architectures on segmenting T1 and T2 MRI images of the neonatal brain according to tissue types. HyperDense-Net performed better than LiviaNET, although it required a longer duration of training. Hong et al. **(ms. 591683)** developed a DL architecture for **segmenting MRI images of the fetal cortical plate during development**. They propose a fully convolutional neural network with a novel hybrid loss function and multi-view (axial, coronal, and sagittal) aggregation using a test-time augmentation, enabling the use of three-dimensional (3D) information. They demonstrate that these methods improve the accuracy of cortical plate segmentation. Closing the section on segmentation, Tan et al. **(ms. 481187) introduce DeepBrainSeg, a convolutional neural network for segmenting optical microscope images**. The classical method for parcellating the brain and the cerebral cortex relies on microscopic differences in neurons' size, density, and cortical myelin content, observed through a microscope. Parcellation is essential for the analysis of brain structures and their functions. DeepBrainSeg incorporates three feature levels to learn local and contextual features in different receptive fields through a dual-pathway convolutional neural network. It has been applied to mouse brains but is likely to obtain similar results if applied to larger brains.

Machine learning and deep learning carry the potential of distinguishing healthy brains and brains with neurological or psychiatric conditions, and diagnosing the conditions. Zhang et al. **(ms. 560709)** ***present A*** **survey on deep learning for neuroimaging-based brain disorder analysis**. They provide an overview of deep learning techniques and popular network architectures, and deep learning methods for computer-aided analysis of Alzheimer's disease, Parkinson's disease, Autism spectrum disorder, and Schizophrenia. They also discuss the limitations of existing studies and present possible future directions. Yamaguchi et al. **(ms. 652987)** take on the challenge of overcoming one of the current limitations. They demonstrate that a **3D convolutional autoencoder applied to structural MRI images of schizophrenia patients can extract features related to schizophrenia without relying on diagnostic labels**. They demonstrate that the proposed auto-encoder extracted features retained information that could predict medication dose and symptom severity in schizophrenia. Feature extraction without using diagnostic labels based on the current diagnostic criteria may lead to the development of alternative data-driven diagnostic criteria and could have a significant contribution to neuroimaging of neurological and psychiatric conditions. Another neurological condition whose early diagnosis and classification into sub-types can inform a decision on treatment is **Subcortical Vascular Cognitive Impairment**. Chen Q. et al. **(ms. 543607)** propose a deep learning solution using 3D attention-based Resnet applied to single T2-weighted FLAIR MRI images. The network only requires inputting the data from a new patient. It achieves high accuracy of classification. It is capable of assisting in diagnosis, leading to early treatment of the different subtypes of sub-cortical ischemia.

Deep learning can also support the evaluation of a semi-continuous measure of the severity of a condition. Along these lines, Finck et al. **(ms. 889808)** improve the estimation of lesion load in multiple sclerosis. They investigate the generalizability of a Generative Adversarial Network (GAN) for synthesizing high-contrast double inversion recovery (DIR) images from lower-contrast T1-weighted and FLAIR MRI images. They also propose the use of uncertainty maps to further enhance their clinical utility. They demonstrate that GAN is capable of synthesizing DIR images with virtual multiple sclerosis lesions that cannot be distinguished from measured real lesions. To this end, they applied an attention module and directed the network's attention toward the lesions. They used data obtained in several imaging centers, thus also demonstrating the generalizability of the model to data obtained in a center whose data were not used for training. The method enhances the automatic counting of multiple sclerosis lesions, which is used as a biomarker of the disease severity and an indicator for the required treatment. Chen M. et al. **(ms. 563097) demonstrate early prediction of measures that are used to diagnose cognitive deficits in very preterm infants**. Up to 40% of very preterm infants (≤32 weeks gestational age) are identified with a cognitive deficit at 2 years of age. Yet, an accurate clinical diagnosis of cognitive deficit cannot be made until 3–5 years of age. Chen et al. obtained diffusion MRI data, computed diffusion-based connectome, and applied transfer learning enhanced deep convolutional neural networks. The performance is superior to that obtained by current methods. Moreover, Chen et al. identified the brain regions most discriminative to the cognitive deficit. The results suggest that deep-learning models can facilitate early prediction of neurodevelopmental outcomes in very preterm infants.

Although deep learning has been increasingly used for neuroimaging image analysis, classification and diagnosis, it has not gained as much ground over standard multivariate pattern analysis (MVPA) techniques in the classification of electroencephalography (EEG). The high dimensionality and large amounts of noise present in EEG data, coupled with the relatively low number of examples (trials) that can be obtained from human subjects are disadvantages for deep learning. **To enable the use of deep learning for MVPA**, Williams et al. **(ms. 491877) present a method of “paired trial classification”** that involves classifying pairs of EEG recordings as coming from the same class or different classes. This makes it possible to significantly increase the number of training examples, through the combinatorics of pairing trials. The final classification is pursued by means of a “dictionary” approach: compare the novel example to a group of known examples from each class. The method can be used as a dataset-specific distance metric that can be extended to novel uses.

Applying deep learning in neuroimaging has become inevitable and this trend is likely to continue in the near future. The studies in this Research Topic show how deep learning can be beneficial to neuroimaging and related modalities across healthy and diseased brains. Combined with recent developments of explainable artificial intelligence and self- or semi-supervised methods, the findings of this Research Topic could be enhanced for even greater impact.

## Author contributions

AS wrote the editorial manuscript. All authors contributed to managing the Research Topic and commented on the editorial manuscript. All authors contributed to the article and approved the submitted version.
